# *KIF1A* variants are a frequent cause of autosomal dominant hereditary spastic paraplegia

**DOI:** 10.1038/s41431-019-0497-z

**Published:** 2019-09-05

**Authors:** Maartje Pennings, Meyke I. Schouten, Judith van Gaalen, Rowdy P. P. Meijer, Susanne T. de Bot, Marjolein Kriek, Christiaan G. J. Saris, Leonard H. van den Berg, Michael A. van Es, Dick M. H. Zuidgeest, Mariet W. Elting, Jiddeke M. van de Kamp, Karin Y. van Spaendonck-Zwarts, Christine de Die-Smulders, Eva H. Brilstra, Corien C. Verschuuren, Bert B. A. de Vries, Jacques Bruijn, Kalliopi Sofou, Floor A. Duijkers, B. Jaeger, Jolanda H. Schieving, Bart P. van de Warrenburg, Erik-Jan Kamsteeg

**Affiliations:** 1 Department of Human Genetics, Radboud university medical centre, Nijmegen, The Netherlands; 2 Department of Neurology, Donders Institute for Brain, Cognition, and Behaviour, Radboud university medical centre, Nijmegen, The Netherlands; 3 Department of Neurology, Leiden University Medical Center, Leiden, The Netherlands; 4 Department of Human Genetics, Leiden University Medical Center, Leiden, The Netherlands; 5 Department of Neurology, Brain Center Rudolf Magnus, University Medical Center Utrecht, Utrecht, The Netherlands; 6 Department of Neurology, Ikazia hospital, Rotterdam, The Netherlands; 7 Department of Clinical Genetics, Amsterdam UMC, Vrije Universtiteit Amsterdam, Amsterdam, The Netherlands; 8 Department of Clinical Genetics, Amsterdam UMC, University of Amsterdam, Amsterdam, The Netherlands; 9 Department of Human Genetics and research Institute GROW, Maastricht University Medical Center, Maastricht, The Netherlands; 10 Department of Genetics, Utrecht University Medical Center, Utrecht, The Netherlands; 11 Department of Genetics, University Medical Center Groningen, University of Groningen, Groningen, The Netherlands; 12 Department of Pediatrics, Skaraborg Hospital, Skövde, Sweden; 13 Department of Pediatrics, The Queen Silvia Children’s Hospital, University of Gothenburg Sweden, Gothenburg, Sweden; 14 Department of Pediatric Neurology, Amsterdam UMC, University of Amsterdam, Amsterdam, The Netherlands; 15 Department of Pediatric Neurology, Radboud University Medical Center, Amalia Children’s Hospital and Donders Institute for Brain, Cognition and Behavior, Nijmegen, The Netherlands

**Keywords:** Molecular biology, Movement disorders

## Abstract

Variants in the *KIF1A* gene can cause autosomal recessive spastic paraplegia 30, autosomal recessive hereditary sensory neuropathy, or autosomal (de novo) dominant mental retardation type 9. More recently, variants in *KIF1A* have also been described in a few cases with autosomal dominant spastic paraplegia. Here, we describe 20 *KIF1A* variants in 24 patients from a clinical exome sequencing cohort of 347 individuals with a mostly ‘pure’ spastic paraplegia. In these patients, spastic paraplegia was slowly progressive and mostly pure, but with a highly variable disease onset (0–57 years). Segregation analyses showed a de novo occurrence in seven cases, and a dominant inheritance pattern in 11 families. The motor domain of KIF1A is a hotspot for disease causing variants in autosomal dominant spastic paraplegia, similar to mental retardation type 9 and recessive spastic paraplegia type 30. However, unlike these allelic disorders, dominant spastic paraplegia was also caused by loss-of-function variants outside this domain in six families. Finally, three missense variants were outside the motor domain and need further characterization. In conclusion, *KIF1A* variants are a frequent cause of autosomal dominant spastic paraplegia in our cohort (6–7%). The identification of *KIF1A* loss-of-function variants suggests haploinsufficiency as a possible mechanism in autosomal dominant spastic paraplegia.

## Introduction

Hereditary spastic paraplegia (HSP) is a neurodegenerative disorder that affects motor control over the lower limbs and the bladder, with an incidence of ~1–10 per 100,000 individuals [1]. In the pure form, patients suffer from lower extremity spasticity and weakness and often from urinary urgency. In complicated forms, HSP may be associated with additional neurologic abnormalities, such as intellectual disability, peripheral neuropathy, ataxia, distal muscle wasting, and optic neuropathy [2]. HSP may follow a (de novo) autosomal dominant, autosomal recessive, X-linked, or mitochondrial inheritance pattern. At this moment, 79 loci and 61 corresponding genes have been associated with HSP [3]. The most common autosomal dominant HSP is spastic paraplegia (SPG) type 4, caused by a variant in the *SPAST* gene (40%), while *SPG7* and *SPG11* variants are the most frequent causes of recessive forms.

Variants in *KIF1A* have been described in three different disorders in OMIM. The first is autosomal recessive hereditary sensory neuropathy IIC (MIM # 614213) that has been described in four families. All families had the same frameshift variant that resides in an alternative transcript (NM_001244008.1/NP_001230937.1), suggesting that absence of that alternative transcript was the cause of hereditary sensory neuropathy IIC [4]. The second is autosomal dominant mental retardation type 9 (MIM # 614255), which is characterized by severe cognitive impairment with spastic paraparesis, axonal neuropathy, epilepsy and/or cerebellar atrophy. At least 17 de novo missense variants affecting the kinesin motor domain of *KIF1A* have been described in this syndrome [5–8]. Functional analyses suggested a dominant-negative effect of these missense variants on the movement of this kinesin along the microtubules. The third disorder associated with *KIF1A* is autosomal recessive SPG type 30 (MIM#610357), characterized by slowly progressive pure HSP with an onset in the first or second decade. Missense variants in the kinesin domain of KIF1A are responsible for the three autosomal recessive SPG30 families described to date [9, 10].

A few reports of autosomal dominant SPG make up for a possible fourth disorder, not yet described in OMIM, associated with *KIF1A* [11–13]. Similar to dominant mental retardation type 9 and recessive SPG30, all currently reported *KIF1A* variants in dominant, pure HSP were also missense variants affecting the kinesin motor domain, with the exception of one variant of uncertain significance.

By exome sequencing, we identified variants in the *KIF1A* gene in 24 out of 347 probands with SPG. Variants are not only missense variants in the motor domain of KIF1A, but also loss-of-function variants outside this domain, expanding the spectrum of variants.

## Subjects and methods

### Editorial policies and ethical consideration

Clinical exome sequencing was approved by the Medical Review Ethics Committee, Region Arnhem–Nijmegen, Number 2011/188. All human subjects provided informed consent for this study.

### Patients

Patients with HSP from The Netherlands (80%) and other European countries (20%) were included in clinical exome sequencing as part of their diagnostic work-up. No restrictions on age at onset or inheritance pattern were made, but there had to be a clinical suspicion of a genetic etiology. All patients were counseled by a clinical geneticist or neurologist. From each family, only one patient (proband) was included for exome sequencing. Candidate variants were verified by Sanger sequencing in affected family members whenever possible.

Previous gene testing in some probands of the families presented here excluded the most common forms of autosomal dominant SPG, types 4 and 3A, caused by variants in the *SPAST* gene (13/24 probands) or the *ATL1* gene (7/24 probands), respectively.

Available clinical and imaging data of the probands and affected family members (30 in total) were systematically collected and reviewed.

### Exome sequencing and data analysis

Exome sequencing was performed as previously described [14]. In summary, capture of exons was done using an Agilent SureSelect Human All Exon 50 Mb Kit (Santa Clara, CA, USA). Sequencing was performed using a LifeTechnologies 5500XL machine (Thermo Fisher, Waltham, MA, USA) or an Illumina Hiseq 2000 or 4000 (San Diego, CA, USA). Read mapping and variant calling were done using LifeScope Life Technologies (Thermo Fisher) for the 5500XL data or BWA (mapping) and GATK (calling) for the Illumina data. A filter for a ‘movement disorders’ gene panel was applied. This panel consists of ∼300 genes implicated in various forms of cerebellar ataxia, HSP, genetic choreas, and other hyperkinetic movement disorders [15] and is available from our website (https://www.radboudumc.nl/en/patientenzorg/onderzoeken/exome-sequencing-diagnostics/exomepanelspreviousversions/movement-disorders). The single nucleotide variant data were filtered by [1] frequency (<5% dbSNP, <1% in house database, gnomAD, EXAC [16]) [2] nucleotide and amino acid conservation and [3] exon/intronic position (−20 till +8). Accession number NM_004321.7 and HGVS were used for nomenclature of variants in the *KIF1A* gene. The likelihood of the disease causing effect of these variants was based on frequencies (absence) in control populations, evolutionary conservation, and segregation analyses. The *KIF1A* variants described in this paper were submitted to LOVD (https://databases.lovd.nl/shared/genes/KIF1A).

Copy number variant (CNV) analyses from the WES data were done as described [17]. Essentially, it was done using CoNIFER (http://conifer.sourceforge.net/) [18]. CNVs with an absolute *Z*-score >1.7 were considered for analysis. To reduce false calls due to potential batch effects, analyses are performed using the most recent samples as controls. CNVs were annotated based on the number of RefSeq exons affected, frequency of CNVs within the cohort, and overlap with disease genes from the movement disorders gene panel. Sanger sequencing was used to confirm the presence of the identified variants if the quality score (from GATK) was not sufficient (<500). In addition, Sanger sequencing was used for segregation analyses and to exclude the possibility of bi-allelic *KIF1A* variants.

## Results

### Genetic findings

**Fig. 1:**
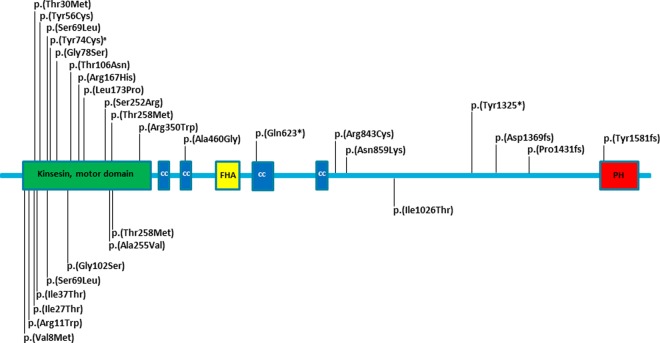
Schematic representation of the KIF1A protein with variants in dominant spastic paraplegia. Variants identified in this study are depicted above the schematic protein, isoform NP_004312.2, while previously described variants in autosomal dominant spastic paraplegia [11–13, 15, 29–32] are shown below the schematic. Protein domains are according to UniProt consortium (https://www.uniprot.org), with kinesin motor, cc coiled coil, FHA forkhead-associated, PH pleckstrin homology domains. ^a^This family has been described [15]

A cohort of 347 probands with a predominantly (>90%) pure SPG was referred to our expert centre for ‘genetic movement disorders’. Clinical exome sequencing was performed as described [14]. Twenty different heterozygous *KIF1A* variants were detected in 24 probands (Table 1 and Fig. 1). One of those variants (in family 24) was a chromosome 2q37 contiguous gene deletion including the entire KIF1A gene (Chr2 [GRCh37]:g.(238475818_238482964)_(243037178_qter)del, Supplemental Fig.). Throughout the remainder of the paper, the variants are discussed by the protein change deduced from their DNA change. Three variants were recurrent, p.(Ser252Arg) in two, p.(Thr258Met) in three, and p.(Arg843Cys) in two families. Targeted sequencing of the *KIF1A* gene was subsequently performed to exclude putative bi-allelic variants, rendering a possible recessive inheritance unlikely. All *KIF1A* variants were absent from an in-house database of >30,000 clinical exomes, and from large sequencing cohorts in gnomAD [16], except for the p.(Arg843Cys) variant that was heterozygously present in one individual in gnomAD. In addition, missense variants were of evolutionary conserved amino acids (in all vertebrates and many even in lower species; Table 1). Two variants, p.(Ser69Leu) and p.(Thr258Met), were reported previously in autosomal dominant HSP [11, 13]. No overlap was seen with the *KIF1A* variants reported in the allelic disorders autosomal recessive hereditary sensory neuropathy IIC, autosomal dominant mental retardation type 9, or autosomal recessive SPG30.

**Table 1:** *KIF1A* variants in autosomal dominant HSP

Family	Variant (heterozygous) DNA; protein	Domain	CADD (PHRED)	Freq in ExAC/gnomAD	Evolutionary conservation	LOVD ID
P1	c.89T>C; p.(Met30Thr)	Motor	26.4	0/0	M, F, DM	00225586
P2	c.167A>G; p.(Tyr56Cys)	Motor	28.6	0/0	M, F, DM, CE	00225587
P3	c.206C>T; p.(Ser69Leu)	Motor	16.3	0/0	M	00246956
P4	c.221A>G; p.(Tyr74Cys)^a^	Motor	25.7	0/0	M, F, DM, CE	00225589
P5	c.232G>A; p.(Gly78Ser)	Motor	34	0/0	M, F, DM, CE	00225590
P6	c.317C>A; p.(Thr106Asn)	Motor	26.3	0/0	M, F, DM, CE	00225591
P7	c.500G>A; p.(Arg167His)	Motor	34	0/0	M, F, DM, CE	00225592
P8	c.518T>C; p.(Leu173Pro)	Motor	27	0/0	M, F, DM, CE	00225593
P9/10	c.756C>G; p.(Ser252Arg)	Motor	25.1	0/0	M, F, DM, CE	00225594 00246690
P11/12/13	c.773C>T; p.(Thr258Met)	Motor	28.5	0/0	M, F, DM, CE	00225610 00246691 00246692
P14	c.1048C>T; p.(Arg350Trp)	Motor	12.95	0/0	M, F, DM, CE	00225611
P15	c.1379C>G; p.(Ala460Gly)	Coiled coil	25.3	0/0	M, F	00225612
P16	c.1867C>T; p.(Gln623*)	ni	38	0/0	ni	00225613
P17/18	c.2527C>T; p.(Arg843Cys)	–	35	1/244238	M, F	00225614 00246693
P19	c.2577C>A; p.(Asn859Lys)	–	28.9	0/0	M, F, DM, CE	00225615
P20	c.3975C>G; p.(Tyr1325*)	ni	29.1	0/0	ni	00225616
P21	c.4096_4103dup; (p.(Asp1369fs)	ni	11.05	0/0	ni	00343920
P22	c.4292del; p.(Pro1431fs)	ni	22.5	0/0	M, F	00225617
P23	c.4740dup; p.(Tyr1581fs)	ni	35	0/0	ni	00225618
P24	Chr2[GRCh37]:g.(238475818_ 238482964)_(243037178_qter)del	ni	ni	ni	ni	00245206

Variants are described according to HGVS nomenclature. Protein domains are according to UniProt consortium (https://www.uniprot.org). Due to nonsense-mediated decay domain of loss-of-function variants are not informative (ni). CADD scores (vs 1.3) and population frequencies were obtained through https://cadd.gs.washington.edu/ and http://exac.broadinstitute.org, respectively. Conservation was determined using ensemble alignments

*M* mammals, *F* fish, *DM* *Drosophila melanogaster*, *CE* C.elegans, *ni* not informative

^a^This family has been described [15]

#### De novo and inherited autosomal dominant KIF1A variants

**Fig. 2:**
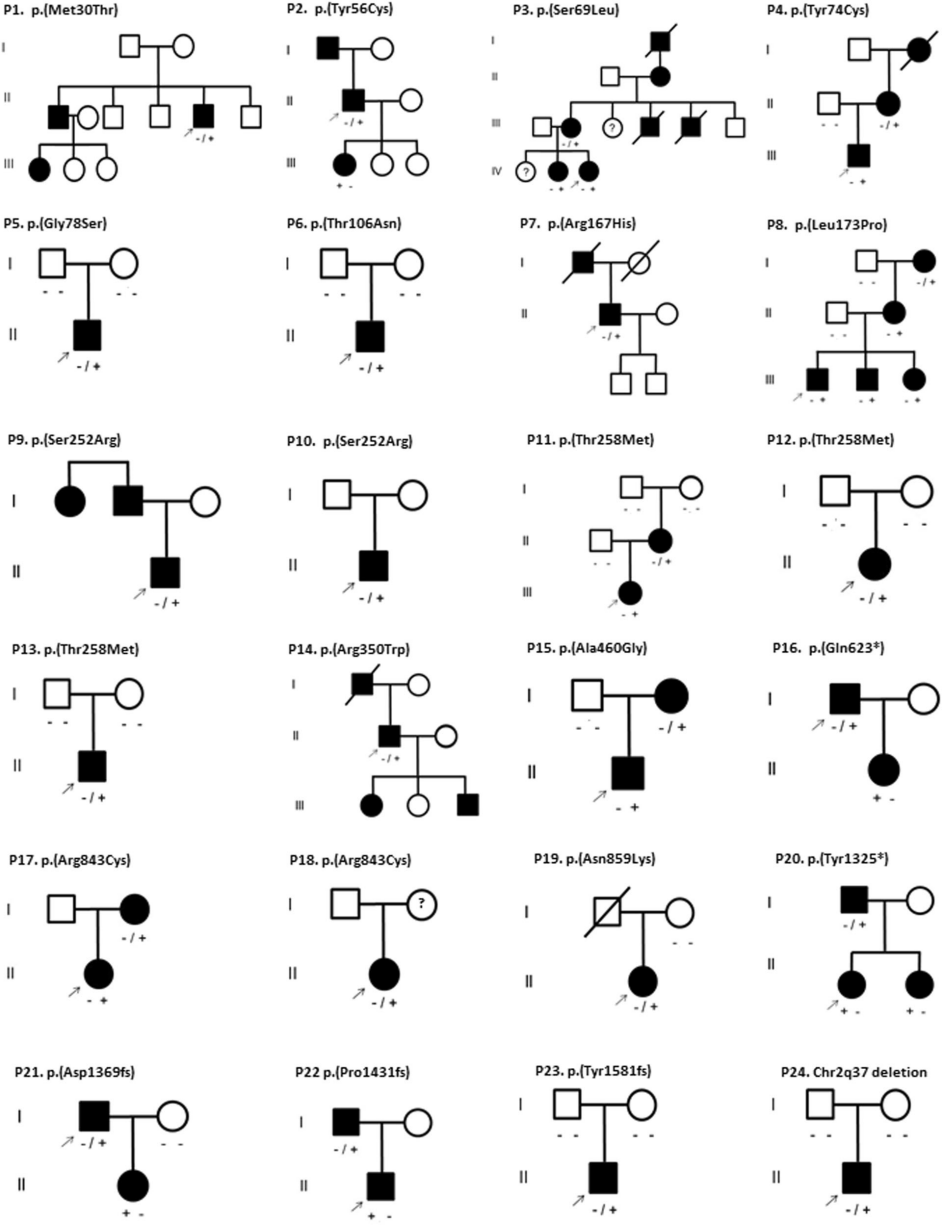
Pedigrees of the 24 families with HSP. Generations are given in Roman numbers, circles are females, squares are males, solid symbols are affected individuals. Deceased individuals are crossed out. Variants are indicated above the pedigree’s by protein nomenclature and indicated by variant (+) and wild type (−) under each tested individual. Arrows indicate the probands. The full HGVS nomenclature of the Chr2q37 deletion is Chr2[GRCh37]:g.(238475818_238482964)_(243037178_qter)del

In 7 of the 24 patients, the *KIF1A* variants (p.(Gly78Ser), p.(Thr106Asn), p.(Thr258Met) in three families, (Tyr1581fs) and the Chr2q37 deletion) had occurred de novo in the first affected generation (Fig. 2; P5, P6, P11, P12, P13, P23, and P24), which was revealed by parental testing including a paternity test. Eleven variants, including one that was de novo in the first generation, were detected in multiple affected family members in multiple generations (P2, P3, P4, P8, P11, P15, P16, P17, P20, P21, and P22), though families usually were small (Fig. 2). In four families (P1, P7, P9, P14), a dominant inheritance was evident, but family members refrained from testing or were deceased. The remaining three probands did not have affected family members and parents were unavailable for testing for de novo occurrence (P10, P18, and P19).

#### Variants in the KIF1A gene in- and outside the kinesin motor domain

**Fig. 3:**
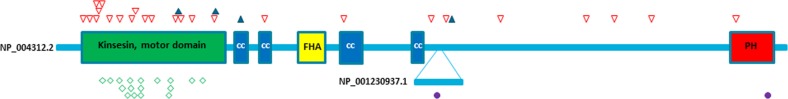
Schematic representation of the KIF1A protein with all variants in the allelic disorders. All variants identified in spastic paraplegia are depicted above the schematic protein in red open triangles (autosomal dominant) or blue solid triangles (autosomal recessive), while variants in mental retardation type 9 and hereditary sensory neuropathy IIC are shown below the schematic in green open diamonds and purple solid dots, respectively. Protein domains are according to UniProt consortium (https://www.uniprot.org)

Variants causing autosomal dominant mental retardation type 9 or SPG30 invariably are missense variants located in the kinesin motor domain of the KIF1A protein (Fig. 3), an important domain for ‘walking’ of the kinesin along microtubules. Functional analyses revealed a dominant-negative effect of these variants in transport along microtubules [5]. Eleven of the twenty variants in this study were also missense variants located in this motor domain (Figs. 1 and 3), suggesting that these variants cause dominant SPG. However, another nine variants were detected outside the motor domain. In contrast to the motor domain variants, six of the variants outside the domain are considered loss-of-function variants (p.(Gly623)*, p.(Tyr1325*), p.(Asp1369fs), p.(Pro1431fs), p.(Tyr1581fs), and the Chr2q37 deletion including *KIF1A*). Two of the loss-of-function variants were shown to be de novo occurrences (P23 and P24) and the four others (P16, P20, P21, and P22) were present in multiple affected family members. Altogether, these data indicate that loss-of function variants in the *KIF1A* gene may also cause autosomal dominant SPG.

Finally, three of the nine variants outside the motor domain were missense variants. In these families, segregation analyses were limited to two affected individuals (P15 and P17) or could not be performed since family members refrained from testing, or were deceased (P18 and P19).

### Clinical findings

The clinical characteristics of 24 probands and six affected family members from families P3, P11, P20, and P21 were analyzed. The overall picture is that of a slowly progressive, pure HSP (Table 2, probands only). Age at onset was highly variable, between 0 and 57 years, although the majority (17/30) presented before age 11 years and only 3/30 after age 40 years. Progression was slow, evidenced by the fact that 25 of 30 patients walked unassisted despite disease durations of over 40 years in some. All had lower limb spasticity accompanied by pyramidal reflex patterns. Deep sensory disturbances in the lower limbs were observed in 11/30, distal and/or proximal leg weakness in 13/30 (accompanied by leg muscle atrophy in some), and sphincter problems were mentioned by 11/30. Arm involvement was seen in 11 patients, but mostly consisted of (slightly) increased tendon reflexes, with occasional increased tone or a postural tremor. Two patients also displayed primitive reflexes. Pes cavovarus (tendency) was observed in 4/30 patients, and spinal deformities (increased lumbar lordosis and thoracic scoliosis) in 3/30. With regard to psychiatric comorbidity, ADHD and depression were both recorded in one patient only. Hearing loss was noticed in one patient. Brain (*n* = 13) and spinal (*n* = 9) imaging was mostly normal, except for thinning of the corpus callosum in two (P12 and P13). Interestingly, this was also observed in the reported family with the same variant p.(Thr258Met) [13].

**Table 2:** Clinical characteristics of probands with KIF1A variants

Patient	P1	P2	P3	P4	P5	P6	P7	P8	P9	P10	P11	P12	P13	P14	P15	P16	P17	P18	P19	P20	P21	P22	P23	P24
Family history	+	+	+	+	−	−	+	+	+	−	+	−	−	+	+	+	+	?	−	+	+	+	−	−
Age at onset (y)	<1	9	13	<10	21	>18	50	<10	57	<10	<10	<1	1	<10	~20	~30	1	46	10	<10	2	2	<5	1
Age (years)	56	14	17	53	38	54	64	50	62	49	18	4	3	54	28	60	19	56	51	66	12	22	20	6, 5
Cognition^a^	N	N	IQ80	N	N	N	N	IQ77	N	N	N	N	N	N	N	N	N	N	N	N	N	N	N	IQ57
Walking aid	−	−	−	+	−	+	+	+	−	−	−	+	−	−	−	−	−	−	−	−	−	−	−	−
LL spasticity	+	+	+	+	+	+	+	+	+	+	+	+	+	+	+	+	+	+	+	+	+	+	+	+
LL weakness	+	+	+	+	+	−	−	+	+	−	−	+	+	−	−	+		−	−	−	+	−	+	+
LL deep sensory disturbances	+	−	+	−	+	−	+	+	−	−	−	+	−	+	−	+		−	−	−	−	−	?	?
PTR	↑	?	↑	↑	↑↑	↑	↑↑	↑↑	↑↑	↑	↑	↑	↑	↑	↑	↑	↑	↑	↑	↑	↑	↑	↑	↑
ATR	↑	↑	↑	↓	↑	↑	↑↑	↑↑	↑↑	↑	↑	↓	↑	↑	↑	↑	↑	↑	↑	↑	↑	↑	↑	↑
Extensor plantar response	+	+	+	+	+	+	+	+	+	+	+	+	+	+	+	+	+	+	+	+	+	+	+	−
UL spasticity	−	−	−	−	−	−	−	−	−	−	−	+	−	−	−	−	+	−	−	−	−	−	−	−
UL weakness	−	+	−	−	−	−	−	−	−	−	−	−	−	−	−	−	?	−	−	−	−	−	−	−
UL sensory disturbances	−	−	−	−	−	−	−	−	−	−	−	−	−	−	−	−	?	−	−	−	−	−	−	?
UL DTR	↑	N	N	↓	N	N	?	N	N	↑	↑	N	N	N	↑	↑	↑	↑	↑	N	N	N	N	N
Urinary sphincter problems	+	+	+	−	+	−	+	+	−	−	+	−	−	+	−	−	−	−	−	−	−	−	−	+
Brain and or spine MRI	?	N	?	?	N	N	?	?	N	?	N	TCC	TCC	?	N	N	N	N	N	?	?	?	N	N

*LL* lower limb, *UL* upper limb, *PTR* patellar tendon reflex, *ATR* Achilles tendon reflex, *DTR* deep tendon reflexes, + present, − absent, *N* normal, ↑ increased, ↓ decreased, *L* left, *R* right, ? no data, *TCC* thin corpus callosum

^a^Not formally assessed in most; based on clinical impression, school performance, and/or current employment

Three exceptions to the phenotype of pure HSP were families P3, P8, and P24. The affected individuals from families P3 and P8 had learning difficulties, with an IQ varying from 68 to 80 points, where IQ below 70 is considered as borderline intellectual disability. In P24, the patient had an intellectual disability (57 IQ points). However, he had a de novo chromosome 2q37 contiguous gene deletion including *KIF1A* and *HDAC4* that very likely explains the more complex phenotype.

## Discussion

In conclusion, we report 24 probands with autosomal dominant SPG with 20 heterozygous variants in *KIF1A*. Three variants were recurrent in our cohort. SPG due to *KIF1A* variants was rather pure. However, two families had cognitive impairment, reflected by learning difficulties and borderline intellectual disability in one patient, whereas one additional proband had a complex phenotype and a de novo contiguous gene deletion including *KIF1A*. Seventeen variants in 20 families are likely to cause dominant SPG, because they are missense variants in the kinesin domain (11 variants, of which 5 had occurred de novo) or are very likely loss-of-function variants outside the motor domain (six variants, of which two were de novo). Three variants, in four probands, are considered to be of uncertain significance and would need further characterization. In addition, all variants were absent or found only once (p.(Arg843Cys)) in controls and affect evolutionary conserved amino acids in alignments of orthologs. The presence of 20 likely disease causing variants (or 24 including the variants of uncertain significance) out of 347 families (6–7%) makes *KIF1A* a frequent cause of autosomal dominant pure SPG.

### Phenotypic differences in KIF1A related disease

Most variants in the allelic disorders associated with KIF1A are located in the kinase motor domain suggesting a common molecular mechanism. The differences in disease severity between autosomal dominant mental retardation type 9, with a severe neurological involvement, and the relative mild HSP in both recessive (SPG30) and dominant forms is therefore intriguing. One explanation may be a difference in the level of impairment of transport by KIF1A.

Kinesins are molecular motors involved in the transport of cargo (e.g., organelles, proteins, mRNA) to their sites of action. The motor domains of kinesins are involved in binding and movement along microtubules, whereas other domains of the kinesins are involved in cargo binding or cargo-induced dimerization [19, 20]. KIF1A is almost exclusively expressed in the brain and spinal cord [21] and RNA-sequencing data of human tissues reveals high expression in cerebral cortex, hippocampus, hypothalamus, cerebellum, caudate, and pituitary gland [22]. KIF1A is involved in anterograde transport of vesicles in neuronal axons [23, 24] and it is hypothesized that mild transport impairment may only affect the long axons to the legs and the bladder, resulting in SPG. In contrast, strongly impaired transport will also affect neurons more proximally, leading to more severe motor deficits, and possibly also to involvement of other neuronal populations that lead to mental impairment [5]. The difference between a dominant and recessive inheritance of SPG due to KIF1A variants may likewise be a result of subtle differences in the efficiency of the delivery of cargo to the periphery of the motor neurons.

Another possibility is that there is some redundancy in the expression of different kinesins, such as the kinesin1, −2, −3, −4, −11, and −13 family members [25]. In affected neuronal populations, some of these other kinesins may possibly compensate for ‘mild’ *KIF1A* variants only. In other brain regions that express *KIF1A* but are unaffected, even in the severe mental retardation type 9, such compensation by other kinesins may be sufficient for normal function. These mechanisms, however, are yet speculative and need further molecular assessments.

Patients of two families had learning difficulties, and one had borderline intellectual disability. Possibly, these mild cognitive impairments may be attributed to the *KIF1A* variants. As such, pure SPG and mental retardation type 9 may be the two extremes of one disease spectrum, where the two families with learning disabilities are intermediates. However, mild cognitive impairment, such as learning difficulties, is relatively common in the general population and was also seen in some family members not having the *KIF1A* variant. More cases with *KIF1A* variants and this intermediate phenotype need to be discovered before a causal relationship may be assigned.

### 2q37 microdeletion syndrome/brachydactyly-mental retardation syndrome

The *KIF1A* gene is located in the cytogenetic 2q37.3 band, and is deleted in a subset of the 2q37 microdeletion syndrome patients. This microdeletion syndrome is characterized by mental retardation (in most), brachydactyly (in 50%), short stature, obesity, hypotonia, characteristic facial appearance, autism spectrum disorder (in 30%), seizures (in 20–35%), and many other congenital anomalies [26]. Inactivating variants in the *HDAC4* gene in patients without the 2q37 microdeletion, but with brachydactyly and mental retardation, suggested that the *HDAC4* gene is the cause of these two features in the 2q37 microdeletion syndrome [27]. Other genes are likely involved in other characteristics of this syndrome. Spasticity has been reported in decipher in one of the patients (ID 250622) having a 2q37 microdeletion involving *KIF1A*, but not in others. This may be due to the underdiagnosis of SPG in children with severe other neurological problems, or due to a masking of SPG due to hypotonia, which is another frequent finding in the 2q37 microdeletion syndrome. In proband P24, the major problem was SPG, although he was also diagnosed with mild mental retardation and later developed seizures, both likely attributable to haploinsufficiency of *HDAC4*. Reversed phenotyping in a large set of patients with the 2q37 microdeletion syndrome may reveal whether SPG is a commonly overlooked feature, or whether the penetrance of SPG in this syndrome is also incomplete.

### Molecular mechanisms of missense variants in the kinesin motor domain

The anterograde transport of KIF1A motor domain mutants in dominant mental retardation type 9 and, to a lesser extent, in SPG30 was previously found to be impaired, and thereby resulted in decreased localization of KIF1A in distal axons [5, 6]. Therefore, it is anticipated that cargo is still bound by KIF1A, but delivered with reduced speed and numbers at the neuronal distal axons, and that this mechanism is more pronounced in dominant mental retardation type 9 than in recessive SPG30 [5, 6].

The subcellular localization of the KIF1A mutants in dominant SPG has not been analyzed. Nevertheless, we speculate that the anterograde transport of motor domain mutants in autosomal dominant SPG (17 motor domain variants of 26 total, Fig. 1) is also impaired. The fact that they cause a milder disease than the variants in dominant mental retardation type 9, suggest that the transport deficit of the dominant SPG mutants is an intermediate between dominant mental retardation type 9 and recessive SPG30, where both copies of the gene are mutated in the latter disorder. The suggestion of a variant-specific ‘dose-effect’ is corroborated by the fact that there is no overlap between the variants found in dominant SPG and those in dominant mental retardation type 9 or recessive SPG30. More specifically, some variants in dominant SPG (p.(Gly102Ser); p.(Arg350Trp); p.(Arg167His); Fig. 1) affect the same amino acid, but result in a different substitution compared with variants in recessive SPG (p.(Arg350Gly)) [9, 10] or dominant mental retardation type 9 (p.(Gly102Asp); p.(Arg167Cys)) [5]. This proposed relation between variants and their respective allelic disorder indicates that the phenotypic differences are caused by the *KIF1A* variants themselves, rather than by other genetic or environmental factors.

### Molecular mechanisms of *KIF1A* loss-of-function variants

Six of the *KIF1A* variants are likely loss-of-function variants (nonsense, frameshift, and a gene deletion (Chr2q37)) located outside the kinesin motor domain. Two of those had occurred de novo, and three others were present in multiple affected family members. The low occurrence of loss-of-function variants in *KIF1A* in controls has led to a ‘loss-of-function intolerance’ (pLI) probability score of 1.00 (on a 0.00–1.00 scale; gnomAD [13]). Moreover, no loss-of-function variants were detected in our in-house database of >30,000 exomes of patients or parents without SPG. Nevertheless, even though the inheritance seems de novo dominant, additional Sanger sequencing of the *KIF1A* gene in these patients was performed to exclude the possibility of a recessive inheritance. Finally, heterozygous *KIF1A* knockout mice develop neuropathy at older age, indicating that *KIF1A* haploinsufficiency leads to a neurological phenotype [28]. Together, these data suggests that the six loss-of-function variants in *KIF1A* are likely the cause of dominant SPG in these families. In contrast to the variants in the motor domain, loss-of-function variants will not result in an impaired transport of KIF1A itself, but rather a reduced expression of KIF1A in neurons due to nonsense-mediated decay of the variant mRNA, or due to complete absence of transcription of the deleted allele. As a consequence, it is anticipated that decreased levels of KIF1A may result in reduced transport of cargo to the periphery of the neuronal axons, not due to slow transport by motor domain mutants, but because of shortage of KIF1A motors.

### Missense *KIF1A* variants outside the kinesin motor domain

Four *KIF1A* variants detected in patients with dominant SPG, three in this report and one described earlier [12], reside outside the motor domain and are more difficult to interpret. These variants are not present in a predicted protein domain, except for p.(Ala460Gly), which may be present in the major coiled-coil domain involved in dimerization of KIF1A [19, 20]. In addition, these variants are substitutions of conserved amino acids and are absent from or extremely rare in large control databases (ExAC and in-house database of >25,000 exomes). Unfortunately, segregation analyses were not sufficient for these four missense variants to prove their role in dominant SPG. Identification of these variants in other individuals or functional characterization of the mutant kinesins transport characteristics may determine their role in SPG in the future. In summary, we identified heterozygous causative variants in the *KIF1A* gene in 6–7% of patients with autosomal dominant SPG. These variants were not only missense variants in the motor domain of KIF1A, as seen in allelic disorders, but also loss-of-function variants outside this domain. Haploinsufficiency is thus a likely pathogenic mechanism for at least some of the *KIF1A* variants in autosomal dominant SPG.

## Supplementary information

Supplemental figure 1

## References

[1] Hedera P. Hereditary Spastic Paraplegia Overview. In: Adam MP, Ardinger HH, Pagon RA, Wallace SE, Bean LJH, Stephens K, et al., editors. GeneReviews®. Seattle WA 1993. 20301682

[2] Fink JK (2014). Hereditary spastic paraplegia: clinical principles and genetic advances. Semin. Neurol..

[3] Parodi L, Fenu S, Stevanin G, Durr A (2017). Hereditary spastic paraplegia: more than an upper motor neuron disease. Rev Neurol..

[4] Riviere JB, Ramalingam S, Lavastre V, Shekarabi M, Holbert S, Lafontaine J (2011). KIF1A, an axonal transporter of synaptic vesicles, is mutated in hereditary sensory and autonomic neuropathy type 2. Am J Hum Genet.

[5] Lee JR, Srour M, Kim D, Hamdan FF, Lim SH, Brunel-Guitton C (2015). De novo mutations in the motor domain of KIF1A cause cognitive impairment, spastic paraparesis, axonal neuropathy, and cerebellar atrophy. Hum Mutat.

[6] Esmaeeli Nieh S, Madou MR, Sirajuddin M, Fregeau B, McKnight D, Lexa K (2015). De novo mutations in KIF1A cause progressive encephalopathy and brain atrophy. Ann Clin Transl Neurol.

[7] Ohba C, Haginoya K, Osaka H, Kubota K, Ishiyama A, Hiraide T (2015). De novo KIF1A mutations cause intellectual deficit, cerebellar atrophy, lower limb spasticity and visual disturbance. J Hum Genet.

[8] Yoshikawa K, Kuwahara M, Saigoh K, Ishiura H, Yamagishi Y, Hamano Y (2019). The novel de novo mutation of KIF1A gene as the cause for Spastic paraplegia 30 in a Japanese case. eNeurologicalSci.

[9] Klebe S, Lossos A, Azzedine H, Mundwiller E, Sheffer R, Gaussen M (2012). KIF1A missense mutations in SPG30, an autosomal recessive spastic paraplegia: distinct phenotypes according to the nature of the mutations. Eur J Hum Genet.

[10] Krenn M, Zulehner G, Hotzy C, Rath J, Stogmann E, Wagner M (2017). Hereditary spastic paraplegia caused by compound heterozygous mutations outside the motor domain of the KIF1A gene. Eur J Neurol..

[11] Ylikallio E, Kim D, Isohanni P, Auranen M, Kim E, Lonnqvist T (2015). Dominant transmission of de novo KIF1A motor domain variant underlying pure spastic paraplegia. Eur J Hum Genet.

[12] Citterio A, Arnoldi A, Panzeri E, Merlini L, D’Angelo MG, Musumeci O (2015). Variants in KIF1A gene in dominant and sporadic forms of hereditary spastic paraparesis. J Neurol.

[13] Cheon CK, Lim SH, Kim YM, Kim D, Lee NY, Yoon TS (2017). Autosomal dominant transmission of complicated hereditary spastic paraplegia due to a dominant negative mutation of KIF1A, SPG30 gene. Sci Rep..

[14] Neveling K, Feenstra I, Gilissen C, Hoefsloot LH, Kamsteeg EJ, Mensenkamp AR (2013). A post-hoc comparison of the utility of sanger sequencing and exome sequencing for the diagnosis of heterogeneous diseases. Hum Mutat.

[15] van de Warrenburg BP, Schouten MI, de Bot ST, Vermeer S, Meijer R, Pennings M (2017). Clinical exome sequencing for cerebellar ataxia and spastic paraplegia uncovers novel gene-disease associations and unanticipated rare disorders. Eur J Hum Genet.

[16] Lek M, Karczewski KJ, Minikel EV, Samocha KE, Banks E, Fennell T (2016). Analysis of protein-coding genetic variation in 60,706 humans. Nature..

[17] Pfundt R, Del Rosario M, Vissers L, Kwint MP, Janssen IM, de Leeuw N (2017). Detection of clinically relevant copy-number variants by exome sequencing in a large cohort of genetic disorders. Genet Med..

[18] Krumm N, Sudmant PH, Ko A, O’Roak BJ, Malig M, Coe BP (2012). Copy number variation detection and genotyping from exome sequence data. Genome Res.

[19] Huo L, Yue Y, Ren J, Yu J, Liu J, Yu Y (2012). The CC1-FHA tandem as a central hub for controlling the dimerization and activation of kinesin-3 KIF1A. Structure..

[20] Soppina V, Norris SR, Dizaji AS, Kortus M, Veatch S, Peckham M (2014). Dimerization of mammalian kinesin-3 motors results in superprocessive motion. Proc Natl Acad Sci USA.

[21] Okada Y, Yamazaki H, Sekine-Aizawa Y, Hirokawa N (1995). The neuron-specific kinesin superfamily protein KIF1A is a unique monomeric motor for anterograde axonal transport of synaptic vesicle precursors. Cell..

[22] Consortium GT (2013). The Genotype-Tissue Expression (GTEx) project. Nat Genet.

[23] Hirokawa N, Nitta R, Okada Y (2009). The mechanisms of kinesin motor motility: lessons from the monomeric motor KIF1A. Nat Rev Mol Cell Biol.

[24] Yonekawa Y, Harada A, Okada Y, Funakoshi T, Kanai Y, Takei Y (1998). Defect in synaptic vesicle precursor transport and neuronal cell death in KIF1A motor protein-deficient mice. J Cell Biol.

[25] Hirokawa N, Tanaka Y (2015). Kinesin superfamily proteins (KIFs): Various functions and their relevance for important phenomena in life and diseases. Exp Cell Res.

[26] Doherty ES, Lacbawan FL 2q37 Microdeletion Syndrome. In: Adam MP, Ardinger HH, Pagon RA, Wallace SE, Bean LJH, Stephens K, et al., editors. GeneReviews®. Seattle WA 1993. 20301337

[27] Williams SR, Aldred MA, Der Kaloustian VM, Halal F, Gowans G, McLeod DR (2010). Haploinsufficiency of HDAC4 causes brachydactyly mental retardation syndrome, with brachydactyly type E, developmental delays, and behavioral problems. Am J Hum Genet.

[28] Tanaka Y, Niwa S, Dong M, Farkhondeh A, Wang L, Zhou R (2016). The molecular motor KIF1A transports the TrkA neurotrophin receptor and is essential for sensory neuron survival and function. Neuron..

[29] Iqbal Z, Rydning SL, Wedding IM, Koht J, Pihlstrom L, Rengmark AH (2017). Targeted high throughput sequencing in hereditary ataxia and spastic paraplegia. PLoS One.

[30] Morais S, Raymond L, Mairey M, Coutinho P, Brandao E, Ribeiro P (2017). Massive sequencing of 70 genes reveals a myriad of missing genes or mechanisms to be uncovered in hereditary spastic paraplegias. Eur J Hum Genet.

[31] Dong EL, Wang C, Wu S, Lu YQ, Lin XH, Su HZ (2018). Clinical spectrum and genetic landscape for hereditary spastic paraplegias in China. Mol Neurodegener.

[32] Erlich Y, Edvardson S, Hodges E, Zenvirt S, Thekkat P, Shaag A (2011). Exome sequencing and disease-network analysis of a single family implicate a mutation in KIF1A in hereditary spastic paraparesis. Genome Res..

