# Supplementary figures and images for: KIF1A variants are a frequent cause of autosomal dominant hereditary spastic paraplegia
Source: Eur J Hum Genet. 2019 Sep 5;28(1):40–9. doi: 10.1038/s41431-019-0497-z (PMC6906463; doi:10.1038/s41431-019-0497-z)

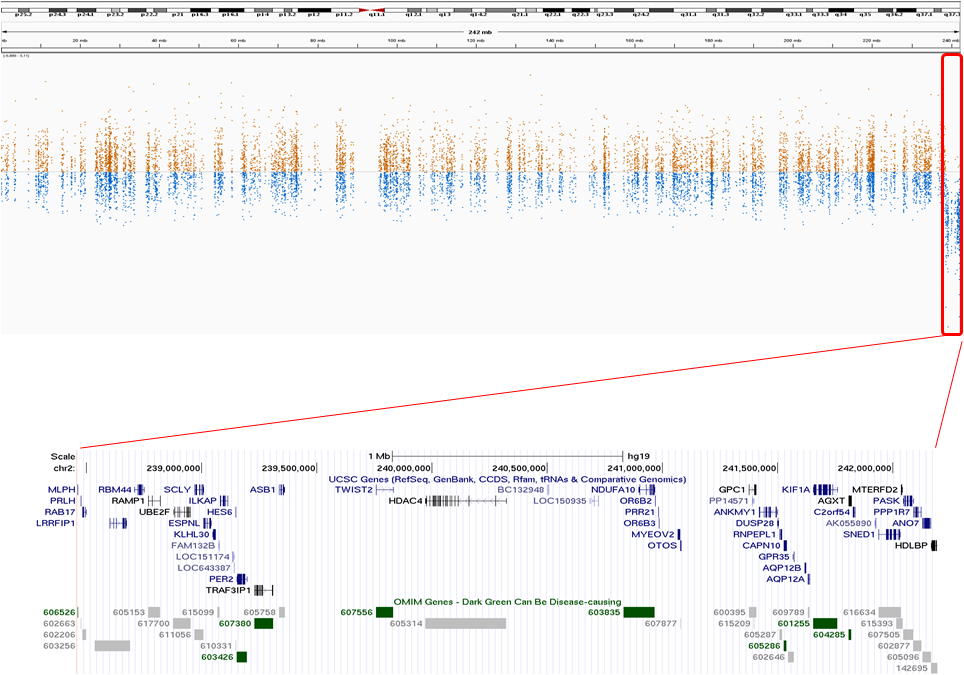

Supplement: Supplementary file 1 — Supplemental figure 1 [file 41431_2019_497_MOESM1_ESM.tif]
